# 

**Published:** 1893-06

**Authors:** 


### CONTENTS FOR JUNE, 1893.

PAGE.

Decennial Address. By Thomas Lothrop, M. D. .... 645

ORIGINAL COMMUNICATIONS.

The Nature of and Choice of Methods in the Treatment of Uterine Cancer. By Z. H. Evans, M. D. .... 653
The Causes and Treatment of Chronic Gonorrhea. By J. Henry Dowd, M. D. .... 660
Iodide of Potassium in Potts' Paraplegia. By Samuel E. Milliken, M. D. .... 665

TRANSLATION.

Constipation and Other Disorders of the Large Intestine; Treatment by Olive Oil Enemata. By G. A. Himmelsbach, M. D. .... 666
Abstracts .... 676
New Instruments .... 683

EDITORIAL.

College Commencements .... 684
An Important Meeting .... 689
The American Surgical Association .... 690
Topics of the Month .... 691
Obituary .... 693
Personal .... 694
Academy of Medicine Notes .... 696
Hospital Notes .... 696
Society Meetings .... 697
Book Reviews .... 702

